# Interactive Algorithms for Teaching and Learning Acute Medicine in the Network of Medical Faculties MEFANET

**DOI:** 10.2196/jmir.2590

**Published:** 2013-07-08

**Authors:** Daniel Schwarz, Petr Štourač, Martin Komenda, Hana Harazim, Martina Kosinová, Jakub Gregor, Richard Hůlek, Olga Smékalová, Ivo Křikava, Roman Štoudek, Ladislav Dušek

**Affiliations:** ^1^Institute of Biostatistics and AnalysesFaculty of MedicineMasaryk UniversityBrnoCzech Republic; ^2^Department of Anesthesiology and Intensive Care Medicine, University Hospital BrnoFaculty of MedicineMasaryk UniversityBrnoCzech Republic

**Keywords:** medical education, patient simulation, algorithms, students, community networks, problem-based learning, serious games, survey

## Abstract

**Background:**

Medical Faculties Network (MEFANET) has established itself as the authority for setting standards for medical educators in the Czech Republic and Slovakia, 2 independent countries with similar languages that once comprised a federation and that still retain the same curricular structure for medical education. One of the basic goals of the network is to advance medical teaching and learning with the use of modern information and communication technologies.

**Objective:**

We present the education portal AKUTNE.CZ as an important part of the MEFANET’s content. Our focus is primarily on simulation-based tools for teaching and learning acute medicine issues.

**Methods:**

Three fundamental elements of the MEFANET e-publishing system are described: (1) medical disciplines linker, (2) authentication/authorization framework, and (3) multidimensional quality assessment. A new set of tools for technology-enhanced learning have been introduced recently: Sandbox (works in progress), WikiLectures (collaborative content authoring), Moodle-MEFANET (central learning management system), and Serious Games (virtual casuistics and interactive algorithms). The latest development in MEFANET is designed for indexing metadata about simulation-based learning objects, also known as electronic virtual patients or virtual clinical cases. The simulations assume the form of interactive algorithms for teaching and learning acute medicine. An anonymous questionnaire of 10 items was used to explore students’ attitudes and interests in using the interactive algorithms as part of their medical or health care studies. Data collection was conducted over 10 days in February 2013.

**Results:**

In total, 25 interactive algorithms in the Czech and English languages have been developed and published on the AKUTNE.CZ education portal to allow the users to test and improve their knowledge and skills in the field of acute medicine. In the feedback survey, 62 participants completed the online questionnaire (13.5%) from the total 460 addressed. Positive attitudes toward the interactive algorithms outnumbered negative trends.

**Conclusions:**

The peer-reviewed algorithms were used for conducting problem-based learning sessions in general medicine (first aid, anesthesiology and pain management, emergency medicine) and in nursing (emergency medicine for midwives, obstetric analgesia, and anesthesia for midwifes). The feedback from the survey suggests that the students found the interactive algorithms as effective learning tools, facilitating enhanced knowledge in the field of acute medicine. The interactive algorithms, as a software platform, are open to academic use worldwide. The existing algorithms, in the form of simulation-based learning objects, can be incorporated into any educational website (subject to the approval of the authors).

## Introduction

Medical education is constantly evolving by gradually, but significantly, shifting from traditional methods (eg, textbooks, lectures, bedside teaching) to a more comprehensive approach that also employs modern information and communication technology (ICT) tools (eg, e-learning, interactive algorithms, computer simulations, virtual patients). Such approaches have been demonstrated to enhance and improve the learning skills of medical students and residents in comparison to traditional methods [1-3]. Several ancillary factors in medicine and medical education have also contributed significantly to these trends; in particular, the rapid development of new technologies and the generally preferred shorter hospital stays, which reduces the student’s exposure to a given case or diagnosis. The economic efficiencies of Web-based education and traditional face-to-face education approaches were compared under randomized controlled trial conditions in Maloney et al [4] and it was shown that the Web-based education approach was clearly more efficient from the perspective of the education provider.

Although most of the modern interactive tools are intended for extending and supplementing the traditional methods rather than replacing them, they have undoubtedly brought a number of advantages, such as equal and easy access for the students to all diagnoses, simulation of a variety of real-life situations, comprehensive interdisciplinary learning, and a higher level of comfort for hospitalized patients. Simulation-based learning also provides the unique opportunity of practicing knowledge application in a manner that mimics real-time patient care without posing a risk to the patient [5,6]. On the other hand, developing simulations and e-learning materials requires investment of the time of skilled professionals (eg, physicians, teachers, programmers); therefore, it is necessary to ensure that the time and resources expended is justified by the educational impact [7]. Furthermore, the developed tools are often accepted uncritically and with emphasis on technological sophistication at the expense of the underlying psychopedagogical theories [1].

Improved efficiency in the development of digital teaching and learning materials, as well as their higher quality, can be achieved by sharing the educational content and by initiating collaborative multi-institutional authoring teams together with joint efforts in establishing the methods for quality evaluation. The management of multisource content among academic institutions brings the necessity of correct indexing, metadescription, and proper categorization [8], as well as reimbursement [9-11] for the created resources. The idea of the medical faculties in the Czech Republic and Slovakia sharing their educational digital contents surfaced in 2006 for the first time. Soon after, in 2007, all 7 Czech medical faculties as well as all 3 Slovak medical faculties formally joined the new network. In 2012, representatives of the Czech and Slovak health care institutions joined the Medical Faculties Network (MEFANET) education network. The MEFANET project [12] aims to develop cooperation among the medical faculties to further the education of medical and health care disciplines using modern ICT via a common platform for sharing digital education content, as well as for assessing their quality through a multidimensional approach [13].

Most of the digital teaching described in recent literature has been prepared as Web-based works because Web technologies allow for easy incorporation of multimedia objects, interactive algorithms, animated simulations, etc. The work may then be easily accessed from any computer and by a defined target audience (eg, students of a particular medical school or course). The developed tools and simulations cover a wide range of medical disciplines, such as critical care [14,15], cardiology [3], hematology [1], neurology [16], surgery [17], metabolic disorders, imaging methods [18,19], and cytogenetics [20].

Acute medicine is a dynamic environment with high demands on team communication and leadership, requiring correct clinical reasoning and quick decision making under time pressure. Simulation offers a good and interesting platform for training multidisciplinary medical teams, facilitating interaction among the team members and enabling the team to function in an effective and coordinated manner [6]. Internet education resources for intensive care medicine have recently been reviewed by Kleinpell et al [14], who demonstrated that most of them are electronic forms of textbooks and articles rather than interactive algorithms and dynamic simulations. Davids et al [7] described an interactive Web-based simulation in which the user treats patients with electrolyte and acid-base disorders, selects the therapies and doses, and can immediately see the treatment results.

In this paper, we present the education portal AKUTNE.CZ [21] as an important part of the MEFANET’s contents. It aims to be a comprehensive source of information and education materials covering all aspects of acute medicine for undergraduate and postgraduate students of the medical and health professions. We focus here primarily on the simulation-based tools for teaching and learning algorithms for acute patient care that form the backbone of AKUTNE.CZ. The simulations take the form of interactive algorithms and represent the basis for a new extension of MEFANET’s activities incorporating focus on serious games.

## Methods

### Overview

MEFANET [12] has established itself as the standard-setting body for medical educators in the Czech Republic and Slovakia, 2 independent countries that once comprised a federation, have similar languages, and still retain the same curricular structure for medical education. One of the basic goals of the network is to advance medical teaching and learning with the use of modern information and communication technologies. As an instrument, MEFANET has decided to develop an original and uniform solution for educational Web portals that are used, together with a central gateway, to offer and share digital education content. Students—approximately 16,500 potential users and academic staff and approximately 3900 potential users from all Czech and Slovak medical faculties—can find their e-learning materials at 11 standalone faculties’ instances of an educational portal with the use of the indexing and searching engine, MEFANET Central Gateway [22].

### MEFANET e-Publishing System

**Figure 1:**
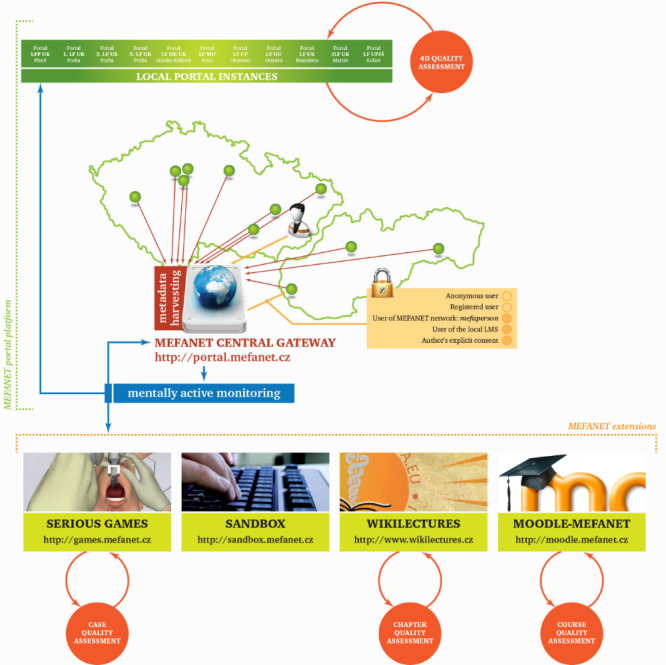
MEFANET involves all medical schools in the Czech Republic and Slovakia. They share one another’s digital teaching and learning materials by using an e-publishing system that consists of 11 educational Web portals and a central gateway. The extensions of the MEFANET e-publishing system appear as standalone platforms for their users. However, all teaching or learning materials indexed by the MEFANET Central Gateway undergo the same procedures of multidimensional quality assessment.

The idea of a shared e-publishing system is based on a set of standalone Web portals rather than on a centralized application hosted for all medical schools, which might be an inflexible and more vulnerable alternative solution. Each portal instance represents an independent publication media with its own International Standard Serial Number (ISSN) code and an editorial board. Local metadata describing the digital educational contents are replicated regularly to the central gateway (see metadata harvesting in Figure 1). There are 3 fundamental elements that have to be rigidly maintained on the part of local administrators: (1) the medical disciplines linker, (2) the authentication/authorization framework, and (3) multidimensional quality assessment. The other features, properties, and functionalities can be adapted or localized to meet the needs of the particular institution. A detailed description of the 3 fundamental elements is as follows. See [13] for full and comprehensive information.

The medical disciplines linker represents the main taxonomy of contributions within the frame of the network. With its single-level list of 56 medical specializations, it forms the only obligatory structure of a portal instance. Any change to its content is subject to approval of the MEFANET Coordinating Committee.

The authors of the shared teaching materials can choose from the following user groups to permit or deny access to their materials: (1) nonregistered anonymous users, (2) registered anonymous users who accept the terms of use within their registration, (3) users of the MEFANET network, that is, a student or teacher from any Czech or Slovak medical school (MEFAPERSON), (4) users from a local university whose affiliation to that university has been verified at the portal via the local information system of that university, (5) users to whom attachments are made available only after the author’s explicit consent. Services of the Czech academic identity federation, eduID.cz [23], are used to check the affiliations of the users of the portal instances. This federation uses the Shibboleth technology, which is one of the several authentication frameworks allowing the sharing of Web resources among institutions using the Security Assertion Markup Language (SAML) protocol standard. The portal instances behave like service providers in this federation, whereas the information systems of the involved schools act as identity providers.

**Figure 2:**
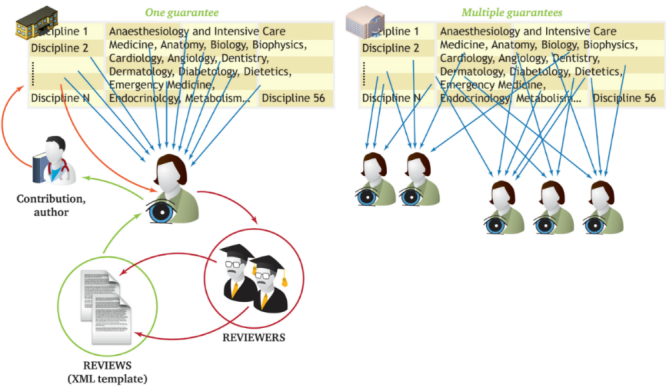
The contribution workflow scheme: (1) the author and technical editor finishes the contribution, (2) the guarantor, who is associated with a particular medical discipline, is notified about a new contribution to his/her field of interest, (3) the guarantor, either alone or with the help of the faculty’s editorial committee, invites 2 reviewers to present their reviews online with the use of template-generated forms.

There are 4 dimensions of critical importance when evaluating the quality of electronic teaching materials: (1) expert review, (2) education level of target users, (3) classification by type, and (4) self-study score. The review includes binary questions as well as open questions. The structure of the review form can be localized by modifying an extensible markup language (XML) template file. The second dimension is represented by the education level of the target group of the teaching material, which is a useful piece of information for the users and the reviewers. The next dimension is represented by a multiple-choice classification according to the types of attachments—the enumerated scale includes static files for Web-based learning and interactive e-learning courses encapsulated in the learning management systems. The last dimension—a self-study score—indicates what users think about the usability of a particular contribution in their self-studies. The values of the first 3 dimensions of the 4D assessment are composed by authors, guarantors, and reviewers. Their activities and the workflow of a contribution are explained in Figure 2. In addition to the 4D quality assessment, all contributions submitted to the central gateway undergo an additional editorial process called *mentally active monitoring*. It focuses on the following issues: (1) metadata is filled in properly, (2) granularity of the attachments is suitable, and (3) all attached documents and the links are accessible for at least MEFAPERSON users. The monitoring of these 3 important issues is done not only at the syntax level, but also semantically; therefore, it is carried out by a team of editors in cooperation with the editors responsible for the local Web portals.

Recently, new tools for technology-enhanced learning have been introduced to the MEFANET network in addition to the common e-publishing portal platform. These new tools complement the portal platform suitably because they provide a higher level of interactivity for students during their self-study process. Figure 1 shows how the new 4 tools—Sandbox [24], WikiLectures [25], Moodle-MEFANET [26], and Serious Games [27]—are related to the already established and standardized MEFANET Central Gateway.

The Serious Games extension is the latest development in MEFANET and it is designed for indexing metadata about simulation-based learning objects, also known as *electronic virtual patients* or *virtual clinical cases*. The first comprehensive set of such interactive learning objects is composed by algorithms for acute patient care published at the AKUTNE.CZ educational portal [21] together with other digital education materials covering a wide range of acute medicine topics.

### Interactive Algorithms for Teaching and Learning Acute Medicine

**Figure 3:**
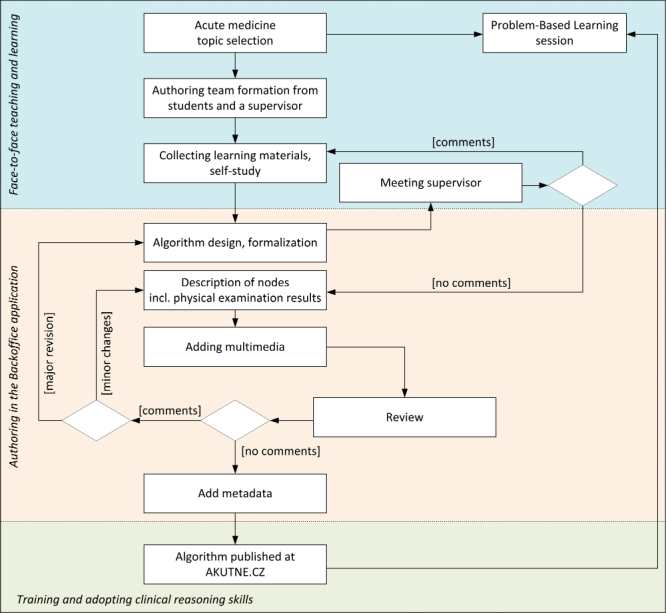
The authoring workflow of an interactive algorithm from choosing the topic through a review process to deployment to teaching in the form of a moderated problem-based learning session.

Each physician dealing with acute patients needs algorithmic thinking and correct clinical reasoning. Our interactive algorithms take the form of content-rich virtual cases because they link together process flowcharts and multimedia. Creating such algorithms or electronic virtual patients is laborious, time-consuming, and often accompanied by ambiguities and hesitations. Following the principles of student-centered learning, our authoring teams consisted of medical students in the final years of their studies, supervised by an experienced clinician. The complete workflow of the authoring process is outlined in Figure 3.

It takes 10 to 50 hours of active work to produce 1 interactive algorithm. The time of the team members is spent on collaborative work, meetings, and on self-studying. Student–authors consult their problems and reservations with a supervisor assigned to them and the resulting product is then submitted to an external reviewer, usually an experienced clinician or an academic staff from another workplace. After the incorporation of all reviewers’ comments, the algorithm is completed by metadata to be published on the AKUTNE.CZ educational portal. Finally, sets of algorithms are compiled together with their metadata into a contribution to be published and indexed on the MEFANET Central Gateway. These contributions with a wider scope than individual algorithms are subjected to the multidimensional quality assessment described previously. Finished and published algorithms are used by other students either as outlines for problem-based learning (PBL) sessions or as supplementary materials for training and adopting correct clinical reasoning.

**Figure 4:**
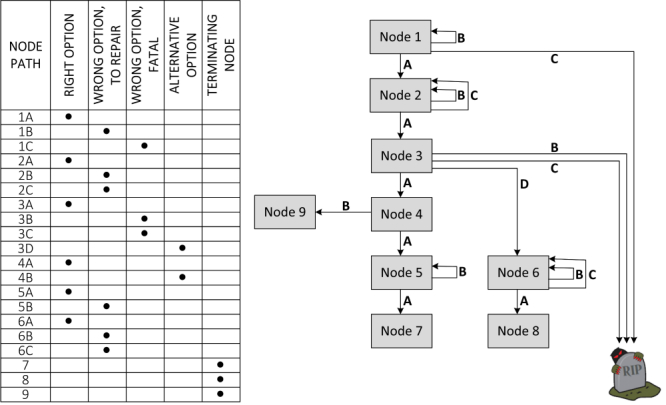
Various types of nodes and options/answers that may be used for authoring an interactive algorithm.

**Figure 5:**
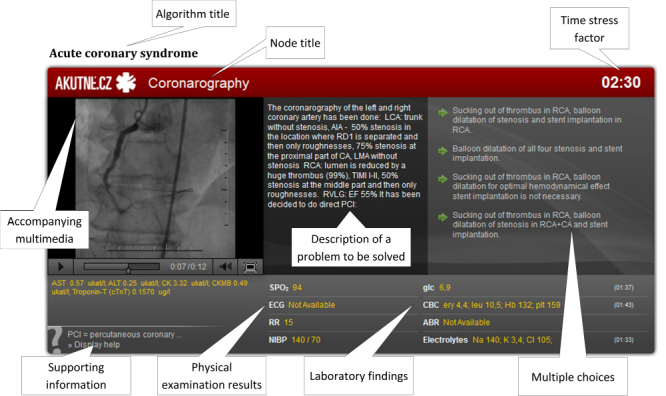
An explained screenshot for 1 node of an algorithm for training clinical reasoning skills in acute coronary syndrome.

The interactive algorithms are authored with the use of a Web-based (PHP/MySQL) BackOffice application that provides the student–authors the following functionalities through its online forms and drag and drop control: (1) node-based scenario design, (2) description of the situation in each node, including the intervals of parameter values of physical examinations, intervals of laboratory values, and multimedia, (3) description of the correct answers as well as distractors with the option to repeat or end in a fatality, and (4) data export for each finished algorithm into an XML document. The XML documents are then rendered into a Flash object resembling a serious game. A student–player uses the game or this simulation-based learning object by moving between the nodes, which may be of different types, as shown in the sample algorithm in Figure 4. Each move causes a shift in the timeline as a side effect of the student–player’s action, lending authenticity to the scenario and creating a stress effect, which is pronounced in real-life situations when dealing with acute patients. Continuous change of various numerical parameters reflecting the development of patient’s clinical status and vital functions in time (eg, blood pressure, pulse, oxygen saturation) is also available (see the example of a node of a selected algorithm in Figure 5).

### Students’ Feedback on the Interactive Algorithms

We asked students about their attitudes and interest in using the interactive algorithms as part of their medical or health care studies. The purpose was to ascertain how the students perceived our efforts on authoring and implementing simulation-based learning tools that are so demanding to create. An anonymous questionnaire of 10 items (see Table 1 for complete overview of questions and answer options) was created and presented via SurveyMonkey [28], a free online survey software. Data collection lasted for 10 days in February 2013. The students who enrolled at 1 of the educational workshops or a conference organized by the group around the AKUTNE.CZ portal were asked to complete the survey. The first 4 questions were aimed at obtaining basic data about the respondents, so that the ones who did not study any field of medicine or health care could be filtered out as well as the ones who did know about our interactive algorithms at all. Further questions were answered with a 5-point Likert scale and 1 binary question was aimed at seeking feedback on the use of our interactive algorithms in the studies of acute medicine topics.

**Table 1:** Questionnaire for collecting the students’ feedback on the interactive algorithms.

#	Question	Answer options
1	State your gender.	Male or female
2	What is your field of study?	General medicine
		Dentistry
		Health care specializations (MSc)
		Health care specializations (BSc)
		Midwifery (BSc)
		Postgraduate doctoral program. another (specify, please)
3	What is your attitude toward the interactive algorithms AKUTNE.CZ?	I do not know what they are
		I know what they are, but I have never used them
		I tried to solve at least 1 interactive algorithm
		I am an author or a coauthor of at least 1 interactive algorithm
4	Have you ever used for your studies a serious game (simulation of real situations for teaching and learning) or-any other interactive algorithm AKUTNE.CZ?	I have not used any at all, not even any interactive algorithm
		No. I have used only the interactive algorithms
		Yes. I have used also...(specify which):
5	The interactive algorithms AKUTNE.CZ are an effective tool for my learning.	5-point Likert scale from strongly disagree to strongly agree
6	The use of the interactive algorithms AKUTNE.CZ improved my knowledge in the field of acute medicine.	5-point Likert scale from strongly disagree to strongly agree
7	The use of the interactive algorithms AKUTNE.CZ represents for me a better way to study than static textbooks.	5-point Likert scale from strongly disagree to strongly agree
8	I like playing the interactive algorithms AKUTNE.CZ not only at home, but also at school under the supervision of teachers, together with consulting possible answers as well as with discussion on all issues related to the topic.	5-point Likert scale from strongly disagree to strongly agree
9	Multimedia accompanying the decision nodes together with the time stressor evokes an authentic atmosphere of clinical reasoning and decision making.	5-point Likert scale from strongly disagree to strongly agree
10	Would you recommend the interactive algorithms AKUTNE.CZ to your friends?	Yes or no

## Results

Over 5 years, almost 25 interactive algorithms in the Czech and English languages have been developed and published on the AKUTNE.CZ educational portal to allow the users to test and improve their knowledge and skills in the field of acute medicine. Another 5 algorithms will be finished during 2013. They cover a wide range of acute medicine topics in the following 5 packages:

### Basic Life Support and Advanced Life Support

Algorithms cover many basic life support (BLS) and advanced life support (ALS) procedures described in the current European Resuscitation Council guidelines. We developed a BLS for adults algorithm, ALS for bradycardia, BLS for choking children, and a foreign-body airway obstruction in adults algorithm.

### Emergency Medicine

Emergency medicine is a very specific type of care in exceptional conditions. We tried to create an ambience of a real car accident in the interactive algorithm. Further topics of emergency medicine are algorithms for water rescue, severe hypothermia in the mountains in winter, out-of-hospital craniocerebral injury, and syncope.

### Critical Care Medicine

Critical care medicine (CCM) is the flagship of medicine in general. It is no coincidence that the most demanding and complex algorithms are from this field. The surviving sepsis algorithm is based on the surviving sepsis guidelines of the Society of Critical Care Medicine (SCCM). The acute coronary syndrome algorithm provides a complete decision tree for a patient with acute myocardial stroke. The algorithm for diabetes mellitus deals with sudden loss of consciousness in a diabetic patient.

### Anesthesiology

These algorithms cover both interesting acute and propaedeutic situations during anesthesia. We developed an algorithm describing the correct approach to the parturient with postdural puncture headache after epidural labor analgesia. Another acute situation is described in the algorithm for toxic reaction to anesthetic agents. Propaedeutic skills are represented by algorithms introducing the insertion of central venous catheter or the choosing of venous entry routes.

### Pain Management

Providing good analgesia for acute and chronic pain is a global issue. We cover these issues with an acute postoperative pain algorithm and by algorithms with correct approach to analgesia in a general practitioner’s and a dentist’s surgery/clinic.

User’s attendance to the interactive algorithms was analyzed with the use of Google Analytics in context of the whole website AKUTNE.CZ within a 1-month period (January 15 to February 14, 2013). In this period, 3342 unique users visited the website (5452 visits in total, 176 visits per day, SD 53.1). All interactive algorithms together had 816 unique users. Of 816 users, 297 (36.4%) accessed the algorithms from Brno and were, therefore, identified as students of the Faculty of Medicine in Brno. Other large groups of visitors were from Prague (99/816, 12.1%) and Bratislava (26/816, 3.2%), both major cities with established medical education facilities. On the other hand, 259 accesses (31.7%) were from places where no faculty of medicine exists. Although we are aware of the limited information value of such analysis (eg, not all visits from Brno are performed at school, or a visitor from a small village could be a student from the Brno faculty of medicine), these results document that the interactive algorithms have been used within the whole MEFANET network and a significant proportion of students use them in places outside of the school (ie, in their homes and during leisure time). The most frequently played algorithms were the diabetes mellitus (94/816 unique users, 11.5%), hypothermia (89 unique users, 10.9%), and surviving sepsis (52 unique users, 6.4%).

**Figure 6:**
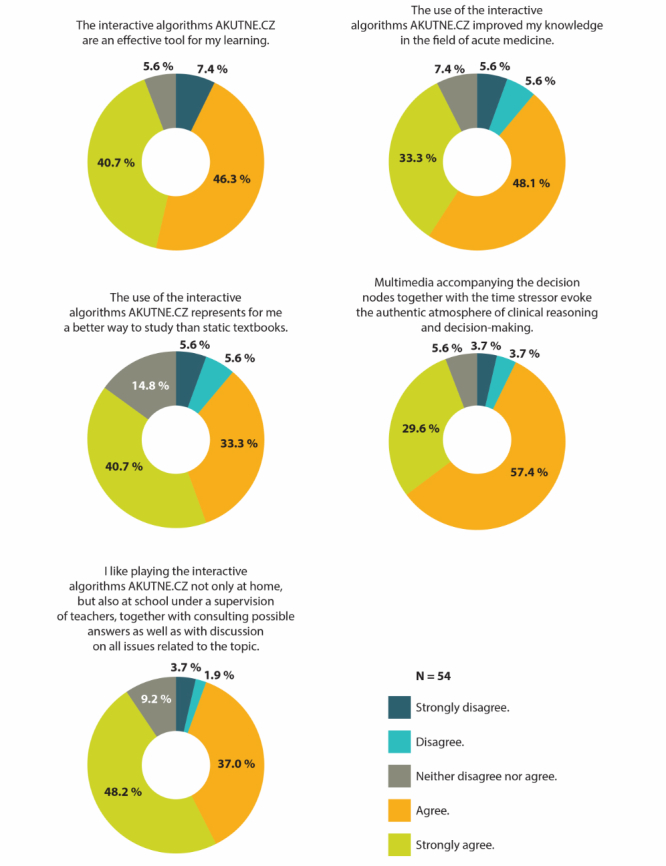
Attitudes and interests of students about using the interactive algorithms as part of their medical or health care studies.

In the feedback survey, 62 participants (13.5%) completed the online questionnaire out of the overall 460 asked to participate. Of all respondents, 66.1% were women and 33.9% were men. After filtering out the participants who were not students of any medical or health care program, and those who did not know about the availability of the interactive algorithms AKUTNE.CZ, the resulting responses from 54 participants were analyzed (see Figure 6). The participants were asked whether the interactive algorithms served as an effective tool for their learning. Four responses were negative or very negative (7.4%), 3 responses were neutral (5.6%), and 47 responses were positive or very positive (87.0%). The participants were further asked whether the interactive algorithms improved their knowledge of acute medicine. Six responses were negative or very negative (11.2%), 4 responses were neutral (7.4%), and 44 responses were positive or very positive (81.4%). In all, 40 participants agreed or strongly agreed (74.0%) that the interactive algorithms represented for them a better study method in comparison to static textbooks, whereas 6 participants disagreed or strongly disagreed (11.2%), and a further 8 respondents neither agreed nor disagreed (14.8%). The participants’ attitude toward interactive algorithms as a tool for face-to-face teaching and learning was positive or very positive in 46 responses (85.2%), negative or very negative in 3 responses (5.6%), and neutral in 5 responses (9.2%). Most participants agreed or strongly agreed (47/54, 87.0%) that multimedia and the time-stress factor provided an authentic atmosphere for pertinent clinical reasoning, whereas 4 participants disagreed or strongly disagreed (7.4%) with this fact and 3 were unsure (5.6%). All participants (100%) stated that the interactive algorithms were worth recommending to their friends.

## Discussion

### Principal Findings

High-quality digital education content production has become a matter of prestige at medical schools in the Czech Republic and Slovakia, and the volume of teaching and learning materials available is growing rapidly thanks to the MEFANET project and its ICT platforms, which have been continuously developed and adopted to the needs of the MEFANET community during the past 6 years. Four new extensions, which complement the e-publishing portal platform standardized in MEFANET, are usable independently; however, their complex application in conjunction with the portal platform as a tool for final e-publishing will allow more effective repurposing of the materials created with the use of the extensions, as well as broader integration of the digital education contents among the MEFANET community. Further development aims to encourage the publication of materials for the teaching of clinical reasoning based on the concept of interactive algorithms or virtual patients. Such simulation-based learning objects are aimed to help the student in developing the much-needed confidence to manage acute conditions, to react accurately, and to avoid distraction by secondary issues.

The unique advantage of interactive algorithms AKUTNE.CZ is the possibility to create complex and branching scenarios. Nevertheless, real-life medical emergencies offer little or no extra options; in many cases, there is only 1 correct course of action. Unfortunately, this feature has not been adopted on a wide scale. The reason could be the characteristics of real-time acute medicine situations that are often linear with no space for branching. On our part, we have complied as much as possible with the guidelines of medical societies. Any deviation from the approved procedures may lead to deteriorating outcomes in real clinical situations. This is the reason why we prefer creating simplified and linear algorithms. An algorithm that approaches realistic simulation (nonlinear or open format) could be more attractive for the students, but we believe that to happen at the expense of didacticism. We also prefer topics that are endorsed and processed by the guidelines or recommendations of the European medical societies (ie, European Resuscitation Council, SCCM, European Society of Regional Anaesthesia and Pain Therapy) and/or national medical societies (ie, Czech Society of Anaesthesiology and Intensive Care Medicine, Czech Society of Intensive Care Medicine, Czech Society of Hematology, Czech Society of Cardiology, Czech Gynecological and Obstetrical Society, and Czech Pain Society). The linear scenarios help to maintain a didactic focus of the interactive algorithms. This mechanistic approach may, however, be detrimental to the students’ understanding of the underlying physiological processes. In order to overcome this limitation, we prefer to use the interactive algorithms for teaching in the form of moderated PBL sessions. Inspired by several works in the field of advanced physiological simulators with a mathematical background [29-31], we will focus our future developments toward a technology mashup, which would allow to incorporate time-dependent, complex physiological simulation of multiple variables and their response to perturbations into the multimedia part of the interactive algorithms.

We cover a wide range of acute medicine topics through the AKUTNE.CZ algorithms. Of course, there is room for additional themes, for example, the widely publicized case of methanol poisoning in 2012 in the Czech Republic, which led to fatalities. Other topics under consideration include selected amyotrophic lateral sclerosis scenarios and out-of-hospital medical emergencies. Interactive algorithms are also used during obstetric anesthesia and analgesia lessons for the midwives—severe peripartal bleeding, amniotic fluid embolism, and out-of-hospital delivery algorithms. The primary aim is to achieve a situation whereby each acute medicine teaching unit has at least 1 interactive algorithm for PBL.

Although the algorithms were tailored to the teaching and learning of acute medicine issues, it is possible to use them for education in other medical and health care disciplines as well. The selection of the parameters from physical examination results and laboratory tests can be changed easily and, thus, adopting the tool for use elsewhere. In comparison with other examples of simulation-based learning objects, such as virtual patients [32], we have a different approach to handling the selected physical examination results and laboratory findings. We follow real-world scenarios and provide the possibility to record these parameters as they are recorded during management of real acute patients too. Each measurement is linked to an increase of the time-stress factor. Thus, students not only learn about dynamics of these characteristics, but also about the unpleasant price in terms of time spent for unnecessary measurements.

A major problem with any medical issue is topicality. AKUTNE.CZ algorithms overcome such problems by ensuring regular updates through the combined efforts of medical students and the authors, in addition to holding regular meetings on time-scheduled updated topics. The algorithms truly reflect on the current medical recommendations and guidelines of the medical societies.

In general, our survey points to a fairly strong preference for the AKUTNE.CZ interactive algorithms by the students as part of their medical or health care studies, although it is notable that the participants were only just aware of the interactive algorithms—a small proportion (9%) reported using other serious games or simulation-based learning objects for their studies. Nevertheless, positive attitudes toward the interactive algorithms outnumbered negative responses. Confirming our expectations, one of the strongest positive answers concerned the participants’ desire to use the interactive algorithms not only for their self-studies during leisure time, but also in face-to-face teaching and learning. Based on our several preliminary attempts at implementing the PBL principles into our teaching, we are fully confident about PBL-like sessions conducted on the node-based scenarios of selected interactive algorithms as the appropriate way to fulfill that wish. The medical and health care institutions in the Czech Republic and Slovakia involved in MEFANET are currently, however, in the very preliminary phases of implementing PBL into their curriculum. Hopefully, the use of interactive algorithms in the process of PBL implementation shall pave the way toward increased attractiveness of our teaching, as well as deeper interest on the part of the students not only in acute medicine issues.

### Limitations

A limitation of the study is that we did not collect data to observe effects of the use of algorithms on expected improvements of participants’ knowledge or on their reactions in real situations. We can only guess about the positive impacts of the interactive algorithms from the fact that most of the student–authors did not have any difficulties launching their professional careers in acute medicine. Another improvement indicator can be inferred from the repeated successes of student–authors and student–players in international competitions of medical rescue teams.

### Conclusions

The methodological aspects of our interactive algorithms for incorporation in the learning and teaching of acute medicine were presented. These interactive algorithms comprise the main part of the educational content of the AKUTNE.CZ portal and recently became the basis for a new extension for MEFANET, the education network of all medical faculties in the Czech Republic and Slovakia.

There are 25 algorithms in the Czech/Slovak and English languages, published online and covering a wide range of topics in acute medicine. The peer-reviewed algorithms were used for conducting PBL-like sessions in general medicine (first aid, anesthesiology and pain management, emergency medicine) as well as in nursing (emergency medicine for midwives, obstetric analgesia and anesthesia for midwives).

We investigated the students’ perception of our interactive algorithms as an adjuvant to their medical and health care studies, especially in relation to clinical reasoning. The feedback from the survey among the AKUTNE.CZ users suggests that the students identify the interactive algorithms as an effective learning tool, serving to enhance their knowledge in the field of acute medicine. In addition, they expressed their keen desire to apply them not only in their leisure time, but also during face-to-face contact with their teachers at school or during clinical practice in the university hospital.

The AKUTNE.CZ interactive algorithms, as a software platform, are open to academic use worldwide. The already created and peer-reviewed algorithms, as simulation-based learning objects, can be included easily into any education website (subject to approval of the authors).

## Abbreviations

ALS: advanced life support
BLS: basic life support
CCM: critical care medicine
ICT: information and communication technology
ISSN: International Standard Serial Number
MEFANET: Medical Faculties Network
PBL: problem-based learning
SAML: Security Assertion Markup Language
SCCM: Society of Critical Care Medicine
XML: extensible markup language
